# Hyperglycemia and risk of ventricular tachycardia among patients hospitalized with acute myocardial infarction

**DOI:** 10.1186/s12933-018-0779-8

**Published:** 2018-10-19

**Authors:** Hoang V. Tran, Joel M. Gore, Chad E. Darling, Arlene S. Ash, Catarina I. Kiefe, Robert J. Goldberg

**Affiliations:** 1 Department of Quantitative Health Sciences, University of Massachusetts Medical School, 368 Plantation Street, Worcester, MA 01605 USA; 2 Department of Internal Medicine, University of Massachusetts Medical School, Worcester, MA USA; 3 Department of Emergency Medicine, University of Massachusetts Medical School, Worcester, MA USA; 4 Department of Medicine, Bridgeport Hospital, Yale New Haven, CT USA

**Keywords:** Glucose, Hyperglycemia, Ventricular tachycardia, Arrhythmia, Myocardial infarction

## Abstract

**Background:**

Little is known about the association of hyperglycemia with the development of ventricular tachycardia (VT) in patients hospitalized with acute myocardial infarction (AMI) which we examined in the present study. The objectives of this community-wide observational study were to examine the relation between elevated serum glucose levels at the time of hospital admission for AMI and occurrence of VT, and time of occurrence of VT, during the patient’s acute hospitalization.

**Methods:**

We used data from a population-based study of patients hospitalized with AMI at all central Massachusetts medical centers between 2001 and 2011. Hyperglycemia was defined as a serum glucose level ≥ 140 mg/dl at the time of hospital admission. The development of VT was identified from physicians notes and electrocardiographic findings by our trained team of data abstractors.

**Results:**

The average age of the study population was 70 years, 58.0% were men, and 92.7% were non-Hispanic whites. The mean and median serum glucose levels at the time of hospital admission were 171.4 mg/dl and 143.0, respectively. Hyperglycemia was present in 51.9% of patients at the time of hospital admission; VT occurred in 652 patients (15.8%), and two-thirds of these episodes occurred during the first 48 h after hospital admission (early VT). After multivariable adjustment, patients with hyperglycemia were at increased risk for developing VT (adjusted OR = 1.48, 95% CI = 1.23–1.78). The presence of hyperglycemia was significantly associated with early (multivariable adjusted OR = 1.39, 95% CI = 1.11–1.73) but not with late VT. Similar associations were observed in patients with and without diabetes and in patients with and without ST-segment elevation AMI.

**Conclusions:**

Efforts should be made to closely monitor and treat patients who develop hyperglycemia, especially early after hospital admission, to reduce their risk of VT.

## Introduction

Ventricular tachycardia frequently complicates the clinical course of patients hospitalized with acute myocardial infarction and is associated with worse in-hospital and long-term outcomes, especially sudden cardiac death [1, 2]. The development of ventricular tachycardia (VT) in patients with an acute myocardial infarction (AMI) is typically associated with a larger infarct, ST segment elevation, left ventricular dysfunction, and more extensive underlying coronary artery disease [3, 4].

During the early stages of AMI, the sympathetic nervous system responds to physiological and emotional stress by releasing noradrenalin, and serum glucose levels typically increase shortly in response to this sympathetic stimulation [5]. The hyperglycemic state can directly alter cardiac electrophysiological status by prolonging the QT-interval, thereby increasing the risk of VT and other serious cardiac arrhythmias [6]. In addition, hyperglycemia is associated with a larger infarct size [7], impaired microvascular circulation [8], and worse left ventricular function [9], which can independently promote electrical instability. Few studies, however, have examined the association between serum glucose levels at the time of hospital admission and occurrence of VT after an AMI [10–12]. Moreover, these studies have been limited by their small sample size, have examined the occurrence of VT with other cardiac arrhythmias which may not share the same underlying mechanisms, or have not examined the time of onset of VT in relation to elevated blood glucose levels [10–13].

Using data from a large population-based surveillance study of patients hospitalized with independently confirmed AMI at all 11 central Massachusetts medical centers, we examined the association between serum glucose levels at the time of hospital admission and the development of VT during the patient’s acute hospitalization.

## Methods

### Study design and data collection

We used data from the Worcester Heart Attack Study, an ongoing population-based clinical/epidemiologic investigation that is examining long-term trends in the incidence, hospital, and post-discharge case-fatality rates of AMI among residents of the Worcester, Massachusetts, metropolitan area hospitalized at all 11 medical centers throughout central Massachusetts for this investigation [14–16]. In brief, the medical records of patients hospitalized for possible AMI at the 11 medical centers serving residents of central Massachusetts were individually reviewed and the diagnosis of AMI was independently validated according to criteria developed by the World Health Organization; patients were further classified into those with an ST-segment elevation AMI (STEMI) or Non-ST segment elevation AMI (NSTEMI) [17]. This investigation was approved by the Institutional Review Board at the University of Massachusetts Medical School.

Trained nurses and physicians abstracted demographic, clinical, and treatment data from the medical records of patients hospitalized with a confirmed AMI [14–16]. Abstracted information included: patient’s age, sex, medical history, physiologic factors, laboratory test results at the time of hospital admission, length of hospital stay, and the in-hospital use of important cardiac medications and procedures (coronary angiography, percutaneous coronary intervention (PCI), and coronary artery bypass graft (CABG) surgery). Development of several significant clinical complications (e.g., atrial fibrillation, cardiogenic shock, stroke, heart failure) during the patient’s index hospitalization were identified using standardized clinical criteria [18–20].

### Ventricular tachycardia

Ventricular tachycardia was defined as a cardiac arrhythmia of three or more consecutive complexes originating from the ventricles at a rate of greater than 100 beats per minute [21]. We included both sustained VT (lasting 30 s or more) and nonsustained VT (lasting less than 30 s) due to similar risk of adverse cardiovascular outcomes associated with both these types of VT [22]. The occurrence of VT was based on physicians’ progress notes and our research physicians also reviewed patients’ hospital ECG strips for purposes of identifying ECG changes consistent with the development of VT. For patients with multiple episodes of VT, only the first episode was counted. Patients were classified as having either early VT, defined as occurring within 48 h after hospital admission, or late (occurring after 48 h) VT.

### Serum glucose levels

Glucose levels were measured from blood samples drawn at the time of admission to the emergency department of all participating medical centers as part of their standard workup of hospitalized patients. All blood tests followed a standardized protocol in the laboratories at each participating hospital.

Prior studies that have examined the prognostic impact of admission serum glucose levels have used different thresholds to define hyperglycemia [23, 24]. For purposes of this study, we defined hyperglycemia as a serum glucose level ≥ 140 mg/dl, the cutoff suggested by the American Heart Association (AHA) for both diabetic and nondiabetic patients [25].

### Study population

There were a total of 5783 patients hospitalized with a verified AMI at all central Massachusetts medical centers on a biennial basis between 2001 and 2011. We excluded from this pool of patients 84 patients who developed VT prior to hospital admission and 18 for whom the timing of VT could not be determined. We also excluded patients with missing data on age (n = 263), race (n = 187), heart rate (n = 100), blood pressure (n = 34), serum potassium (n = 42), glucose (n = 87), white blood cell count (n = 20), troponin I (n = 349), and serum calcium (n = 450) findings, as well as length of hospital stay (n = 9). The final study sample consisted of 4140 patients with an independently confirmed AMI.

### Data analysis

The baseline characteristics of patients who had serum glucose levels greater or less than 140 mg/dl were compared using Chi Square tests for categorical variables and the t-test or Kruskal–Wallis test for continuous variables. We used multivariable logistic regression modelling to examine the association between serum glucose levels at the time of hospital admission with the occurrence of VT, while controlling for several potentially confounding demographic (age, sex, race) and clinical factors (past medical history, clinical presentation at admission, complications during hospitalization, and in hospital treatments). We a priori controlled for age, sex, and race in all regression models. Other variables were then iteratively tested and variables which changed the estimates of the effects of elevated serum glucose levels on the development of VT by more than 10% were retained in the final regression models. After following these predetermined rules, we included age, sex, race, history of diabetes, type of AMI (STEMI vs. NSTEMI), and the in-hospital development of heart failure in our multivariable adjusted models. We also carried out two subgroup analyses in which we examined the association between hyperglycemia and VT in patients with a STEMI in comparison to those with an NSTEMI, and according to a history of diabetes as recorded in hospital medical records. Finally, we examined the association between serum glucose levels and VT in a dose response manner beginning at a serum glucose level of 120 mg/dl and increasing in increments of 20 mg/dl.

## Results

### Baseline patient characteristics

The average age of the study population was 70 (± 13.8) years, 58.0% were men, and 92.7% were non-Hispanic whites. The mean and median [inter quartile range (IQR)] serum glucose levels at the time of hospital admission were 171.4 mg/dl and 143.0 [116.0–203.0] mg/dl, respectively. Hyperglycemia, defined as a serum glucose level ≥ 140 mg/dl at the time of hospital admission, was present in 51.9% of hospitalized patients.

Patients with elevated serum glucose levels at the time of hospital admission for their independently confirmed AMI were approximately 5 years older and were more likely to be female than patients with serum glucose levels < 140 mg/dl (Table 1). Patients with hyperglycemia were more likely to have been previously diagnosed with several important comorbidities (e.g., heart failure, chronic lung disease, chronic kidney disease, diabetes, and hypertension); these patients also had higher heart rate, serum potassium, and serum white blood cell count findings at the time of hospital presentation than patients with glucose levels < 140 mg/dl (Table 1). These patients were also more likely to have developed clinically significant in-hospital complications (e.g., acute kidney injury, atrial fibrillation, heart failure, and cardiogenic shock) and were more likely to have received ACE-I/ARBs and anti-arrhythmic agents during their acute hospital stay compared with patients with lower serum glucose levels. On the other hand, patients with hyperglycemia had lower systolic blood pressure and serum calcium findings at hospital presentation and were less likely to have received aspirin or lipid lowering agents or to have undergone a PCI during their index hospitalization.

**Table 1:** Characteristics of patients according to serum glucose levels at the time of hospital admission for an acute myocardial infarction

Characteristics	< 140 mg/dl (n = 1949)	≥ 140 mg/dl (n = 2191)	p-value
Age (median [IQR], years)	69 [57–81]	74 [63–82]	*< 0.001*
Age (%)			*< 0.001*
< 55	20.7	11.9	
55–64	20.4	16.3	
65–74	20.2	23.9	
≥ 75	38.8	48.0	
Men (%)	62.0	54.4	*< 0.001*
White (%)	93.0	92.5	0.58
Medical history (%)			
Coronary artery disease	32.6	42.9	*< 0.001*
Chronic lung disease	15.6	19.2	*0.002*
Chronic kidney disease	16.5	24.3	*< 0.001*
Diabetes	16.3	53.7	*< 0.001*
Heart failure	17.1	28.1	*< 0.001*
Hypertension	70.3	79.6	*< 0.001*
Hypercholesterolemia	61.8	62.6	0.59
Stable angina	13.9	15.3	0.23
Stroke/transient ischemic attack	12.2	16.0	*< 0.001*
Findings at admission			
Pulse (median [IQR], beat/min)	78 [67–92]	88 [74–104]	*< 0.001*
Systolic blood pressure (median [IQR], mmHg)	141 [122–161]	140 [119–161]	0.18
Troponin I (median [IQR], ng/ml)	0.51 [0.10–3.50]	0.50 [0.10–3.70]	0.72
Potassium (median [IQR], mmol/l)	4.1 [3.8–4.5]	4.2 [3.9–4.7]	*< 0.001*
Calcium (median [IQR], mg/dl)	9.1 [8.7–9.5]	9.0 [8.7–9.4]	*0.002*
WBCC (median [IQR], 10^9^ cell/l)	8.9 [7.2–11.2]	10.4 [7.9–13.4]	*< 0.001*
ST segment elevation	29.8	31.8	0.16
In hospital complications (%)			
Acute kidney injury	10.4	19.3	*< 0.001*
Atrial fibrillation	16.7	22.1	*< 0.001*
Cardiogenic shock	2.4	6.5	*< 0.001*
Heart failure	25.9	45.5	*< 0.001*
Treatment during hospitalization (%)			
Aspirin	94.4	91.6	*< 0.001*
ACE-I/ARBs	67.7	73.3	*< 0.001*
Antiarrhythmic agents	14.6	18.9	*< 0.001*
Beta blockers	92.3	90.9	0.10
Lipid lowering agents	77.2	74.1	*0.018*
Thrombolytic therapy	2.3	2.4	0.74
Percutaneous coronary intervention	51.6	42.0	*< 0.001*
CABG	7.1	6.9	0.80
Length of stay (median [IQR], days)	4 [2–6]	4 [3–7]	*< 0.001*

Italic values indicate significance of p value (*p* < 0.05)

*IQR* interquartile range, *WBCC* white blood cell count, *ACE-I/ARBs* angiotensin converting enzyme inhibitor/angiotensin II receptor blockers, *CABG* coronary artery bypass graft surgery

p-value from Chi square test and Fisher’s exact test for categorical variables and Wilcoxon rank-sum test for continuous variables

### Serum glucose levels and incidence of ventricular tachycardia

In our patient population, VT occurred in 652 patients (15.8%); for two-thirds of these patients (n = 434) their first episode occurred within 48 h of hospital admission. Overall, after adjusting for several demographic and clinical variables of prognostic importance, patients admitted with serum glucose levels ≥ 140 mg/dl had an almost 50% higher odds of developing VT during their acute hospital stay than those with lower glucose levels (Table 2). This association was observed in the 434 patients who developed early VT (adjusted OR = 1.39, 95% CI = 1.11–1.73) whereas among the 218 patients whose VT developed at a later time (more than 48 h after hospital admission), the association between hyperglycemia and late onset VT was weaker and not statistically significant (adjusted OR = 1.19, 95% CI = 0.89–1.59). Adjusting for the hospital receipt of various cardiac interventions and medications did not materially affect the observed associations.

**Table 2:** Serum glucose levels at the time of hospital admission for acute myocardial infarction and development of ventricular tachycardia (VT)

Type of VT	VT (IR)	Odd ratio (95% confidence interval)			
		Unadjusted		Multivariable adjusted	
		< 140 mg/dl	≥ 140 mg/dl	< 140 mg/dl	≥ 140 mg/dl
All episodes of VT	652 (15.7%)	Reference	*1.28 (1.08–1.51)*	Reference	*1.48* ^a^ *(1.23–1.78)*
Early onset VT	434 (10.5%)	Reference	1.21 (0.99–1.48)	Reference	*1.39* ^b^ *(1.11–1.73)*
Late onset VT	218 (5.32%)	Reference	*1.42 (1.07–1.87)*	Reference	1.19^c^ (0.89–1.59)

Italic values indicate significance of p value (*p* < 0.05)

Early onset VT was defined as VT that occurred within 48 h after hospital admission

*IR* incidence rate

^a^Adjusted for age, sex, race, and history of diabetes

^b^Adjusted for age, sex, race, history of diabetes, and AMI type

^c^Adjusted for age, sex, race, and in-hospital development of heart failure

A history of diabetes was present in 36.1% of study patients. As expected, the mean and median [IQR] serum glucose levels were 224 mg/dl and 209 [147–283] mg/dl among patients with previously diagnosed diabetes and 142 mg/dl and 129 [110–157] mg/dl among patients without a history of diabetes. However, the development of VT during the patient’s acute hospitalization was similar among patients with and without a history of diabetes (14.5% vs. 16.5%, respectively, p = 0.09); most events of VT in these patient populations occurred during the first 48 h of hospital presentation (61.6% and 69.0%, respectively, p = 0.06).

In examining the relationship between hyperglycemia and VT further stratified according to the presence of previously diagnosed diabetes, patients with elevated serum glucose levels had a 40–70% higher odds of developing any VT or early VT compared with patients with lower serum glucose levels in those with and without a history of diabetes (Table 3).

**Table 3:** Serum glucose levels at the time of hospital admission for acute myocardial infarction (AMI) and development of ventricular tachycardia (VT) in select patient subgroups

Population	VT/n	Odd ratio (95% Confidence interval)			
		Unadjusted		Multivariable adjusted|	
		< 140 mg/dl	≥ 140 mg/dl	< 140 mg/dl	≥ 140 mg/dl
Diabetes status					
Diabetic	216/1494	Reference	*1.66 (1.11–2.47)*	Reference	*1.72 (1.15–2.57)*
Nondiabetic	436/2646	Reference	*1.36 (1.10–1.67)*	Reference	*1.41 (1.14–1.74)*
AMI type					
STEMI	281/1277	Reference	1.17 (0.90–1.53)	Reference	*1.32 (1.00–1.73)*
NSTEMI	371/2863	Reference	*1.33 (1.07–1.66)*	Reference	*1.30 (1.04–1.63)*

Italic values indicate significance of p value (*p* < 0.05)

*STEMI* ST segment-elevation myocardial infarction, *NSTEMI* non-ST segment-elevation myocardial infarction, *n* subsample

|Adjusted for age, sex, and race

Serum glucose levels were essentially similar for the 1277 patients with STEMI and for the 2863 patients with an NSTEMI. The mean and median [IQR] serum glucose levels were 168 mg/dl and 144 [120–189] mg/dl, respectively, in patients with a STEMI and 173 mg/dl and 143 [114–210] mg/dl, respectively, in patients who had an NSTEMI (p = 0.46). However, VT developed significantly more often in patients with a STEMI than an NSTEMI (22.0% vs. 13.0%, respectively, p < 0.001) and a significantly greater proportion of VT episodes occurred during the first 48 h of hospital admission in patients with a STEMI (78.7%) than among those with an NSTEMI (57.4%), (p < 0.001). Compared with patients who had lower serum glucose levels, patients who had elevated serum glucose levels had an approximate 30% higher multivariable adjusted odds of developing VT, regardless of the type of AMI (Table 3).

Lastly, we carried out an additional analysis in which we examined the association between baseline hospital serum glucose levels and the risk of developing VT, early VT, and late VT at varying serum glucose cut-offs, separately in patients with and without previously diagnosed diabetes (Table 4). As seen, patients with higher serum glucose levels were at greater risk for developing VT and its timing than patients with lower serum glucose levels, irrespective of a history of previously diagnosed diabetes, though there were inconsistencies in the risks observed according to baseline levels of serum glucose.

**Table 4:** Risk of developing ventricular tachycardia (VT) during hospitalization for acute myocardial infarction according to hospital admission serum glucose levels and history of diabetes

Serum glucose levels (mg/dl)	Diabetes previously diagnosed (+) % developing				Diabetes not previously diagnosed (−) % developing			
	n	All episodes of VT	Early onset VT	Late onset VT	n	All episodes of VT	Early onset VT	Late onset VT
< 120	185	9.7	6.5	3.2	996	13.7	9.8	3.8
120–139	132	10.6	6.1	4.6	636	16.7	11.0	5.7
140–159	129	20.9	14.0	7.0	382	17.5	12.6	5.0
160–179	136	22.8	14.7	8.1	235	20.9	13.2	7.7
> 180	912	13.8	8.2	5.6	397	19.7	13.6	6.1

## Discussion

We found that patients with elevated serum glucose levels at the time of hospital admission for an AMI were at considerably greater risk for developing VT during their hospitalization, most notably early during their acute hospital stay, than patients with normoglycemia. This association was observed in both diabetic and nondiabetic patients and in patients with a STEMI and an NSTEMI.

### Serum glucose levels and the risk of developing VT

Elevated serum glucose levels at the time of hospital admission for AMI have been associated with higher death rates in both diabetic and nondiabetic patients [26]. However, supportive evidence for an association between hyperglycemia and developing a serious cardiac arrhythmia, especially VT, is limited. In our large community-based study we found an elevated risk for developing VT among AMI patients with serum glucose levels ≥ 140 mg/dl.

Our findings are consistent with those of a limited number of prior studies, despite their small sample sizes [21], non-optimal reperfusion treatment [10, 11], and failure to examine the relation between hyperglycemia and the time of onset of VT [10, 12]. In a study of 1258 consecutive patients with AMI admitted to a single coronary care unit in Valencia, Spain over a 4-year study period, patients with elevated serum glucose levels at the time of hospital admission had more than twice the odds of developing either VT or ventricular fibrillation than patients with lower serum glucose levels [10].

Several mechanisms have been proposed to explain the potentially proarrhythmic effects of hyperglycemia in patients hospitalized with an AMI. Hyperglycemia can cause QT-interval prolongation and dispersion [27], which can trigger ventricular arrhythmias in those with underlying coronary artery disease [28]. Hyperglycemia has been associated with a larger infarct size, worse left ventricular function [26, 29], and increases in serum biomarkers of inflammation [25, 29] each of which may promote the development of secondary cardiac arrhythmias. Hyperglycemia following an ischemic injury may also be a proxy of increased sympathetic activity, which could exert its proarrhythmic effects through elevated circulatory catecholamines and free fatty acids [30].

Irrespective of the underlying mechanisms involved, the greater risk of developing VT among AMI patients with elevated glucose levels emphasizes the need for systematically assessing serum glucose levels at the time of hospital admission and for more aggressive surveillance and treatment of patients with elevated glucose levels to reduce their risk of developing VT.

### Association of elevated serum glucose levels and timing of VT

After adjusting for several potentially confounding variables, we found that patients with hyperglycemia were at increased risk for developing early VT. Early VT may be triggered by increased sympathetic activity or the inflammatory response to acute ischemic injury, each of which have been shown to be associated with elevated serum glucose levels in patients with AMI [31]. Inflammatory responses to the acute ischemic insult predominately occur during the first few days after an AMI and are associated with increased resting membrane potential and prolonged action potential duration [25, 29], which may contribute to the triggering of early onset VT [32].

While coronary reperfusion therapy may induce reperfusion-associated cardiac arrhythmias, and may have contributed to some of the cases of early VT observed in the present investigation, adjusting for the receipt of coronary reperfusion therapy did not materially change the estimated association of hyperglycemia with the risk of developing VT.

Hyperglycemia was also associated with a higher likelihood of developing late VT, a condition that usually occurs due to reentry pathways via isolated bundles of surviving myocytes at the border of the infarct [33]. Late VT may also occur secondarily to developing heart failure or cardiogenic shock [26], which may be more prevalent in patients with a larger acute infarct, that is also associated with the hyperglycemic state [34].

In the present study, the elevated risk for developing late VT in patients with hyperglycemia was modestly attenuated after adjusting for heart failure and other potentially confounding factors of prognostic importance. These findings suggest that hyperglycemia may act by worsening left ventricular function to promote the onset of late VT. Inasmuch, efforts to reduce infarct size and improve left ventricular function may reduce the late proarrhythmic effects of hyperglycemia.

### Risk of VT in diabetic and nondiabetic patients with elevated serum glucose levels

At the time of hospital admission, patients with diabetes had higher serum glucose levels than patients without this metabolic disorder. However, these higher average glucose levels did not translate into a greater risk for developing VT. Several prior studies have also reported that, compared to patients without diabetes, patients with diabetes had a similar risk of developing new onset ventricular arrhythmias [10, 35].

In separate analyses for patients with and without previously diagnosed diabetes, we found that serum glucose levels ≥ 140 mg/dl at admission were associated with an increased risk of developing VT. Our results are consistent with the findings in the large Korea Acute Myocardial Infarction Registry-National Institutes of Health (KAMIR-NIH) registry of 12,625 patients with AMI [2]. In this registry, the risk of developing VT was higher among patients who had glucose levels > 200 mg/dl at the time of hospital admission than patients who had lower serum glucose levels [36]. On the other hand, the aforementioned study in Spain, similar associations were observed only among patients without, but not those with, a history of diabetes [10]. However, the authors of that study used a liberal cutoff of 180 mg/dl to define hyperglycemia and the risk of VT primarily increased when serum glucose levels were higher than 120 mg/dl in patients with diabetes. Inasmuch, high risk patients might have been included in the two comparison groups [10].

Elevated sympathetic activity and acute inflammatory processes may play a larger role in inducing VT after AMI rather than the effects of chronic high concentrations of glucose itself. Analysis of the Hyperglycemia: Intensive Insulin Infusion In Infarction (HI-5) study showed that the ratio of serum glucose levels at the time of hospital admission, to average glucose levels during the prior 24 months, which also took into account patients prior history of diabetes, was associated with a higher risk of developing serious cardiac arrhythmias [37]. This hypothesis may also help to explain the significant association that we observed between hyperglycemia and the development of early, but not late, VT when sympathetic activation and the inflammatory response may subside. It is also possible that the antidiabetic therapies that patients with a history of diabetes might have received could have prevented or mitigated the onset of VT. However, these hypotheses remain speculative and warrant further investigation.

### Risk of VT in patients with STEMI and NSTEMI with elevated serum glucose levels

Consistent with the results of prior studies [38], we found that VT occurred more frequently in patients with a STEMI than in those with an NSTEMI (22% vs. 13%). This may be due to the complete blockage of a coronary artery that occurs in patients with a STEMI and the subsequent development of hemodynamic disturbances and myocardial electrical instability. In the present study, when the study sample was further stratified according to the presence of the type of AMI, patients with hyperglycemia experienced a 30% elevated odds of developing VT during their acute hospitalization. We are not aware of any previous study that has examined the relationship between elevated serum glucose levels and the risk of developing VT according to the two principal phenotypic expressions of AMI. The increased risk of VT associated with hyperglycemia suggests that close monitoring and treatment of hyperglycemia may reduce the risk of VT in patients with who are hospitalized with either a STEMI or an NSTEMI.

### Study strengths and limitations

The present study has several strengths. We used data from a large, population-based, investigation of patients hospitalized with AMI at all medical centers in central Massachusetts, all of whom were telemonitored for their entire hospital stay, thereby minimizing the possibility of undiagnosed asymptomatic VT. However, some limitations of our observational study need to be acknowledged. The in-hospital management of patients with hyperglycemia may affect the development of VT, especially late VT; however, we were unable to examine the impact of anti-hyperglycemic treatment since we did not collect data on the use of insulin or other antidiabetic medications. We did not collect any information on changes in serum glucose levels during hospitalization for AMI, and, therefore, could not characterize the persistence of hyperglycemia throughout a patient’s acute hospitalization. We also did not collect information about patient’s serum glycated hemoglobin levels for purposes of examining the effects of acute versus chronic hyperglycemia on the development of VT. Lastly, we did not collect information about the sustainability of VT. Inasmuch, we could not examine the relationship between hyperglycemia with sustained and nonsustained VT separately.

## Conclusions

Patients with elevated serum glucose levels were at increased risk for developing VT, especially within the first 48 h after hospital admission, among patients hospitalized with validated AMI at all medical centers in central Massachusetts. Further studies are needed to more fully understand both the biologic mechanisms involved in the associations observed and the effects of hospital therapies that reduce serum glucose levels on the risk of developing VT following an AMI.

## Abbreviations

AHA: American Heart Association
AMI: acute myocardial infarction
CABG: coronary artery bypass graft
IQR: inter quartile range
PCI: percutaneous coronary intervention
NSTEMI: non-ST segment elevation AMI
STEMI: ST segment elevation AMI
VT: ventricular tachycardia
